# GUDCA drives colorectal cancer progression via ALKBH5-mediated m6A modification of ENO1 and glycolytic reprogramming

**DOI:** 10.3389/fphar.2026.1791014

**Published:** 2026-07-10

**Authors:** Ruirong Lin, Shangkun Jin, Weijin Yang, Jiarong Zhang, Jiantao Jiang, Wenmin Xiao, Yun Xie, Changhua Zhuo, Xiaopeng Wang

**Affiliations:** 1 Department of Thoracic Oncology Surgery, Clinical Oncology School of Fujian Medical University, Fujian Cancer Hospital, Fuzhou, China; 2 College of Chemistry, Fuzhou University, Fuzhou, China; 3 Department of Colorectal Surgery, Clinical Oncology School of Fujian Medical University, Fujian Cancer Hospital, Fuzhou, China; 4 Department of Gastric Surgery Oncology, Clinical Oncology School of Fujian Medical University, Fujian Cancer Hospital, Fuzhou, China

**Keywords:** ALKBH5, colorectal cancer, ENO1, glycolysis, GUDCA, m6A modification

## Abstract

**Introduction:**

Colorectal cancer (CRC) is the third most diagnosed malignancy worldwide, yet the mechanisms by which bile acids drive metabolic reprogramming remain unclear. This study investigates whether glycoursodeoxycholic acid (GUDCA) promotes CRC progression through mA epitranscriptomic regulation.

**Methods:**

Serum bile acids were profiled in 106 CRC patients and 106 controls by UHPLC-MS/MS. Cellular phenotypes were assessed by CCK-8, colony formation, and xenograft models. mA modification was mapped by meRIP-seq and validated by meRIP-qPCR. Glycolytic function was measured by lactate production, glucose uptake, and ECAR. FXR binding to the ALKBH5 promoter was assessed by luciferase assay and ChIP-qPCR. Clinical correlations were analyzed in 25 CRC patients undergoing ^1^F-FDG-PET.

**Results:**

GUDCA was the most significantly elevated bile acid in CRC patients and independently predicted poor survival (HR = 3.28, p < 0.001). GUDCA promoted CRC cell proliferation and xenograft tumor growth. Mechanistically, GUDCA antagonized FXR to suppress ALKBH5 transcription, elevating mA modification of ENO1 mRNA and enhancing its translation efficiency. ALKBH5 deficiency drove glycolytic reprogramming and mTORC1 activation. ENO1 silencing abolished GUDCA-induced glycolytic enhancement. Serum GUDCA positively correlated with ^1^F-FDG-PET SUVmax (r = 0.68, p < 0.001). Low ALKBH5 expression correlated with poor patient survival.

**Conclusion:**

GUDCA functions as an oncometabolite that drives CRC progression via the GUDCA–FXR–ALKBH5–ENO1 axis, establishing a feed-forward metabolic circuit linking bile acid signaling to mA-mediated glycolytic reprogramming. These findings identify GUDCA and ALKBH5 as potential therapeutic vulnerabilities in CRC.

## Introduction

Colorectal cancer (CRC) represents the third most diagnosed malignancy and the second leading cause of cancer-related mortality worldwide, with over 1.9 million new cases and 935,000 deaths annually (Bray et al., 2024). Despite advances in surgical techniques and drug therapies, the five-year survival rate for metastatic CRC remains below 15%, indicating an urgent need to identify novel therapeutic targets (Eng et al., 2024). Metabolic reprogramming, particularly enhanced aerobic glycolysis, has emerged as a hallmark of CRC, enabling cancer cells to meet bioenergetic and biosynthetic demands for rapid proliferation (Pavlova et al., 2022; Fendt, 2024).

Bile acids, traditionally recognized for its role in lipid digestion and absorption, have gained growing interest as signaling molecules that modulate glucose homeostasis, lipid metabolism, and cellular proliferation (Collins et al., 2023; Fogelson et al., 2023). Emerging evidence suggest that aberrant bile acid metabolism plays a role in gastrointestinal carcinogenesis, with secondary bile acids promoting DNA damage, inflammation, and cellular transformation (Cai et al., 2022). However, the specific bile acid species driving CRC progression and their molecular mechanisms remain poorly defined.

N6-methyladenosine (m6A), the most prevalent internal RNA modification in eukaryotic mRNAs, dynamically regulates gene expression by modulating RNA splicing, subcellular localization, stability and translation (Roundtree et al., 2017). The m6A modification is catalyzed by methyltransferases (METTL3/14) and reversed by demethylases including fat mass and obesity-associated protein (FTO) and alkB homolog 5 (ALKBH5) (Frye et al., 2018). Aberrant m6A modification has been implicated in various cancers, in which methylases exhibiting context-dependent oncogenic or tumor-suppressive functions (Gu et al., 2020; Wang et al., 2022). Recent studies demonstrate that ALKBH5-mediated m6A demethylation influences cancer cell proliferation, metastasis, and therapeutic resistance in various tumor types (Yang et al., 2023; Chen et al., 2025; Zhai et al., 2023). However, whether bile acids regulate m6A modification to orchestrate metabolic reprogramming in CRC still remains unexplored.

Enolase 1 (ENO1), a glycolytic enzyme catalyzing the conversion of 2-phosphoglycerate to phosphoenolpyruvate, exhibits oncogenic function beyond its metabolic function (Li Z. et al., 2025; Zhang et al., 2024). Aberrant expression of ENO1 correlates with poor prognosis in multiple cancers. Meanwhile, ENO1 overexpression promotes tumor growth, invasion, and metastasis (Fu et al., 2015; Li H. J. et al., 2021). Notably, ENO1 serves as a metabolic vulnerability in glycolysis-addicted cancers, as its inhibition has demonstrated therapeutic efficacy in preclinical models (Sun et al., 2023). Despite growing interest in ENO1 as a potential therapeutic target, the upstream regulatory mechanisms modulaling its expression, particularly at the post-transcriptional level, remain incompletely understood.

In this study, we systematically investigated bile acid profiles in CRC patients and identified GUDCA as a key oncometabolite. We demonstrate that GUDCA drives CRC progression by antagonizing FXR-mediated ALKBH5 transcription, thereby elevating ENO1 m6A modification and translation efficiency. This GUDCA-ALKBH5-ENO1 axis enhances glycolytic and activates mTORC1 signaling, establishing a metabolic circuit that fuels CRC growth. Our findings reveal a novel bile acid-epitranscriptome-metabolism nexus with therapeutic implications for CRC management.

## Results

### Elevated GUDCA levels predict poor prognosis in CRC patients

**FIGURE 1:**
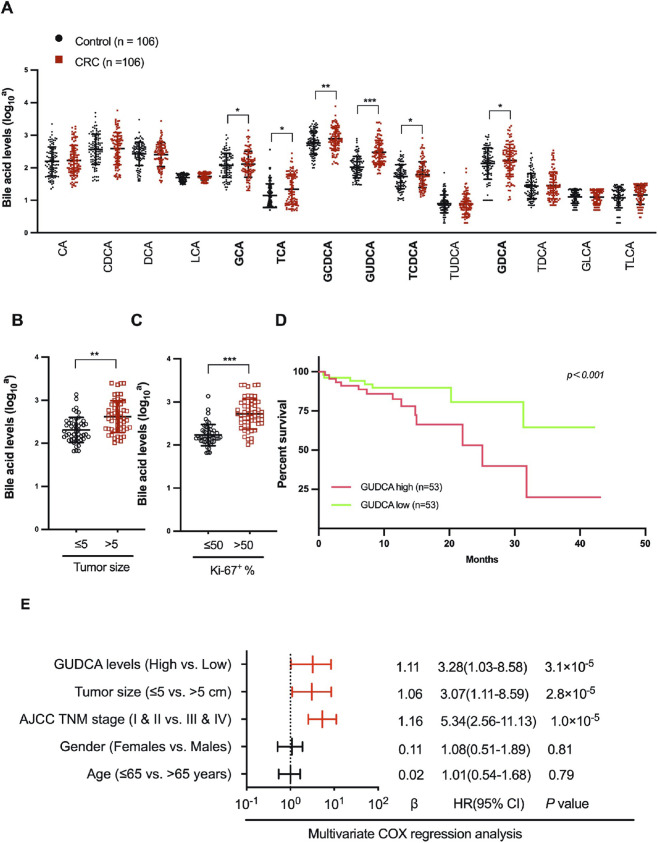
GUDCA Levels Correlate with Tumor Burden and Serve as an Independent Prognostic Indicator in Colorectal Cancer. **(A)** Serum bile acid profiles in 106 CRC patients and 106 healthy controls by UHPLC-MS/MS. Six conjugated bile acids (GCA, TCA, GCDCA, GUDCA, TCDCA, GDCA) were significantly elevated in CRC patients, with GUDCA showing the most significant increase. **(B)** Serum GUDCA levels stratified by tumor size. **(C)** Serum GUDCA levels stratified by Ki-67 proliferation index. **(D)** Kaplan-Meier survival curves for CRC patients with high (n = 53) versus low (n = 53) GUDCA levels. p < 0.001, log-rank test. **(E)** Multivariate Cox regression analysis showing elevated GUDCA as an independent prognostic factor (HR = 3.28, 95% CI: 1.03–8.58, ***p < 0.001). Data are mean ± SD. *p < 0.05; **p < 0.01; ***p < 0.001.

To investigate the role of bile acids in CRC pathogenesis, we performed comprehensive bile acid profiling using ultra-high-performance liquid chromatography-tandem mass spectrometry (UHPLC-MS/MS) in serum samples from 212 individuals (106 CRC patients and 106 healthy controls). Among the bile acid species analyzed, six conjugated bile acids—glycocholic acid (GCA), taurocholic acid (TCA), glycochenodeoxycholic acid (GCDCA), glycoursodeoxycholic acid (GUDCA), taurochenodeoxycholic acid (TCDCA), and glycodeoxycholic acid (GDCA)—were significantly elevated in CRC patients (Figure 1A)**.** Notably, GUDCA exhibited the most significantly increase, indicating that it might serve as a potential biomarker for CRC.

Furthermore, correlation analyses revealed that elevated serum GUDCA levels significantly associated with larger tumor size (>5 cm, p < 0.01, Figure 1B) and higher proliferation index (Ki-67 ≥ 50%, p < 0.001, Figure 1C). In addition, Kaplan-Meier survival analysis demonstrated that CRC patients with high GUDCA levels exhibited significantly reduced overall survival compared to those with low GUDCA levels (Figure 1D). Multivariate Cox regression analysis, adjusting for age, gender, tumor stage, and differentiation grade, confirmed that elevated GUDCA independently predicted poor prognosis (hazard ratio [HR] = 3.28, 95% confidence interval [CI]: 1.03–8.58, p < 0.001; Figure 1E). Although the modest event count per variable (EPV = 6.2) limits the precision of the estimate—reflected in the relatively wide 95% CI—comprehensive model diagnostics ruled out structural overfitting. All variance inflation factors (VIF) were less than 1.5, confirming the absence of multicollinearity. Moreover, the model achieved stable mathematical convergence, and the full-model likelihood-ratio test remained statistically significant (χ2 = 4.8, P = 0.03), demonstrating the overall robustness and stability of the prognostic model. These findings suggest that GUDCA may serve as a clinically relevant oncometabolite in CRC.

### GUDCA promotes CRC Cell proliferation and tumorigenesis

**FIGURE 2:**
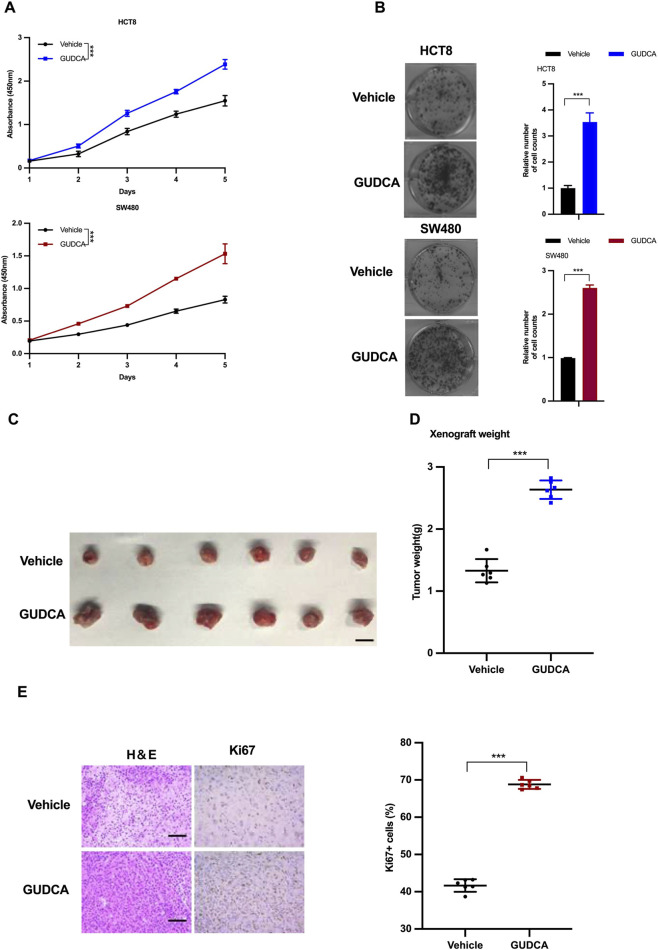
GUDCA Promotes Colorectal Cancer Growth *In Vitro* and *In Vivo*. **(A)** CCK-8 viability assays in HCT8 and SW480 cells treated with GUDCA (50 μM). vs. vehicle. **(B)** Colony formation assays in HCT8 and SW480 cells treated with vehicle or GUDCA (50 μM). Representative images and quantification. **(C)** Tumor growth curves of HCT8 xenografts in mice fed control or 0.1% GUDCA diet (n = 6/group). two-way ANOVA. **(D)** Representative tumor images and quantification of tumor volume and weight at day 28. **(E)** Representative IHC and quantification of Ki-67 in tumor sections. Scale bars = 100 μm. Data are mean ± SD. *p < 0.05; **p < 0.01; ***p < 0.001.

Several recent studies have shown that various BAs play critical roles to fine-tune procession in many type cancers (Režen et al., 2022; Huang et al., 2025). To investigate whether GUDCA impacts CRC progression, we treated human CRC cell lines HCT8 and SW480 with 50 μM GUDCA, a concentration chosen based on plasma levels observed in CRC patients in a previous study (Kühn et al., 2020). GUDCA significantly enhanced CRC cell viability (Figure 2A). Colony formation assays confirmed that GUDCA significantly increased clonogenic potential in both CRC cell lines (Figure 2B).

To validate these findings *in vivo*, we established subcutaneous xenograft models by injecting HCT8 cells into immunodeficient BALB/c nude mice, followed by daily followed by feeding a diet containing 0.1% GUDCA or vehicle control. GUDCA treatment significantly accelerated tumor growth, resulting in increased tumor volume and weight (Figures 2C,D). Immunohistochemical analysis revealed elevated Ki-67 expression in GUDCA-treated tumors, confirming enhanced proliferation (Figure 2E). These results demonstrate that GUDCA exerts potent oncogenic effects in CRC both *in vitro* and *in vivo*.

### GUDCA enhances glycolytic metabolism in CRC cells

Bile acids function as signal-like molecules and play pivotal roles in regulating glucose metabolism, lipid metabolism, and energy homeostasis. Consequently, they have emerged as promising therapeutic targets for various metabolic disorders, including obesity, diabetes mellitus, steatohepatitis, and atherosclerosis (Fogelson et al., 2023; Cai et al., 2022). To investigate the underlying mechanisms, we treated HCT8 cells with GUDCA and performed metabolomic profiling combined with whole-exome sequencing (WES) analysis.

**FIGURE 3:**
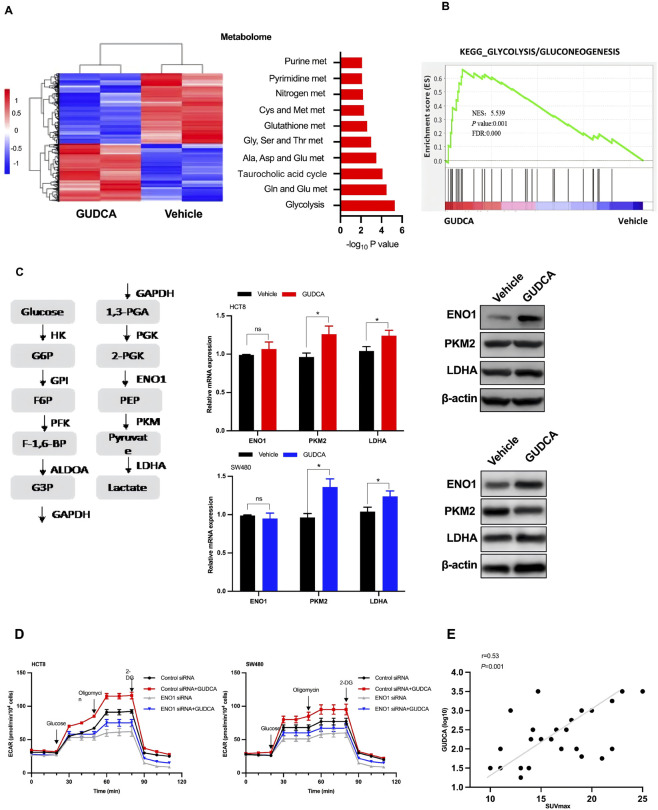
GUDCA-Induced Glycolytic Reprogramming in CRC Cells. **(A)** Metabolomic pathway enrichment analysis and heatmap of glycolytic metabolites in HCT8 cells treated with GUDCA. Top pathways: glycolysis/gluconeogenesis, aspartate/glutamate metabolism, and Taurocholic acid cycle. **(B)** GSEA plot showing enrichment of glycolysis genes in GUDCA-treated HCT8 cells (NES = 5.539, FDR < 0.001). **(C)** Western blots, qRT-PCR, and quantification of ENO1, PKM2, and LDHA in HCT8 and SW480 cells treated with GUDCA. GUDCA selectively increased ENO1 protein without affecting mRNA. **(D)** Glucose uptake (2-NBDG), lactate production, ATP levels, and ECAR profiles in cells treated with vehicle, GUDCA, or GUDCA + siENO1. ENO1 knockdown abolished GUDCA-induced glycolytic enhancement. **(E)** Correlation between serum GUDCA and 18F-FDG-PET SUVmax in CRC patients (r = 0.53, p = 0.001). Data are mean ± SD. *p < 0.05; **p < 0.01; ***p < 0.001; ns, not significant.

Untargeted metabolomic profiling of HCT8 cells treated with GUDCA revealed significant alterations in 68 metabolites (38 upregulated, 30 downregulated). Pathway enrichment analysis identified glycolysis/gluconeogenesis, aspartate and glutamate metabolism, and the Taurocholic acid cycle as the top three dysregulated pathways. Among the glycolysis pathway, the most significantly altered metabolites were phosphoenolpyruvate (PEP), followed by pyruvate and lactate (Figure 3A).

Whole-transcriptome sequencing (RNA-seq) of GUDCA-treated cells corroborated these findings, showing significant enrichment of glycolysis-related gene expression (Figure 3B).

Augmented glycolysis is one of the key drivers of tumorigenesis and progression, and represents a major hotspot in recent research (Paul et al., 2022). Given that PEP, pyruvate, and lactate are the most significantly altered metabolites in the glycolytic pathway, and considering that ENO1, PKM2, and LDHA are the key enzymes responsible for the generation of PEP, pyruvate, and lactate, respectively (Li J. et al., 2025; Wang et al., 2024),we then examined the expression of ENO1, PKM2, and LDHA. Interestingly, GUDCA selectively increased ENO1 protein levels without affecting its mRNA expression, while PKM2 and LDHA remained unchanged (Figure 3C), suggesting post-transcriptional regulation of ENO1.

Functional glycolytic assays confirmed that GUDCA enhanced glucose uptake, lactate production, and ATP generation in CRC cells (Figure 3D). Extracellular acidification rate (ECAR) measurements analysis demonstrated increased glycolytic capacity and glycolytic reserve in GUDCA-treated cells (Figure 3D). Then, we silenced ENO1 using siRNA, which completely abolished GUDCA-induced glycolytic enhancement (Figure 3D), demonstrating that ENO1 is essential for GUDCA-driven metabolic reprogramming. Furthermore, analysis of 25 CRC patients undergoing 18F-fluorodeoxyglucose positron emission tomography (18F-FDG-PET) revealed a positive correlation between serum GUDCA levels and maximum standardized uptake values (SUVmax) (r = 0.68, p < 0.001, Figure 3E), providing clinical validation of GUDCA’s glycolytic role. Together, these findings establish a link between GUDCA and glycolytic activity in colorectal cancer, implicating its role in regulating this metabolic pathway.

### GUDCA suppresses ALKBH5 expression through FXR antagonism

**FIGURE 4:**
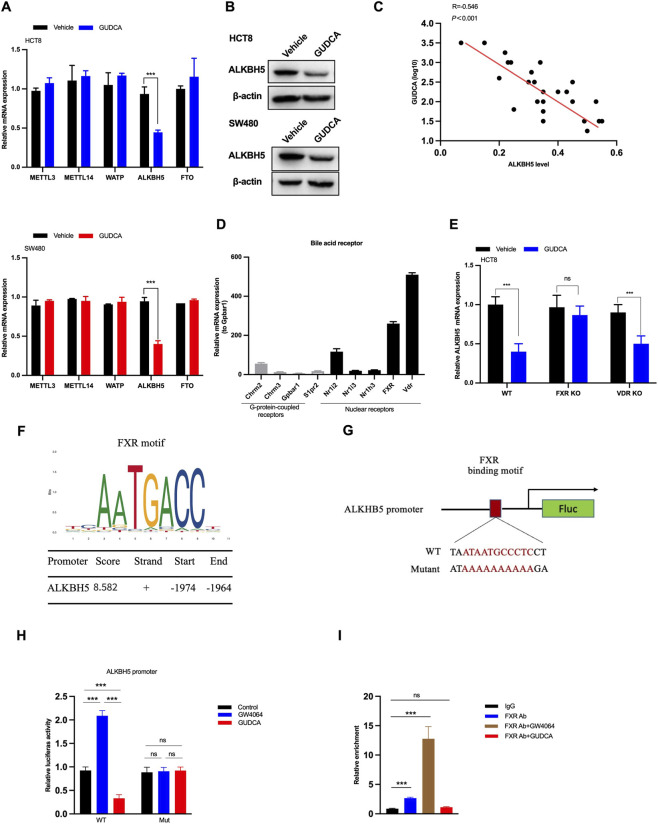
GUDCA Antagonizes FXR to Suppress ALKBH5 Expression in Colorectal Cancer. **(A)** qRT-PCR showing several m6A/dem6A enzymes (METTL3, METTL14, WTAP, FTO) expression in GUDCA-treated HCT8 cells. ALKBH5 was the most downregulated enzyme. **(B)** Western blots demonstrating ALKBH5 suppression by GUDCA in HCT8 and SW480 cells. **(C)** Inverse correlation between serum GUDCA and ALKBH5 protein in CRC tissues (r = −0.546, p < 0.001). **(D)** qRT-PCR of bile acid receptors in HCT8 cells showing FXR and VDR as most highly expressed. **(E)** Western blots showing FXR-KO, but not VDR-KO, abolished GUDCA-induced ALKBH5 suppression. **(F)** Schematic of ALKBH5 promoter with FXR binding sites. **(G)** Sequences of wild-type and mutant FXR binding sites. **(H)** Luciferase assays showing FXR-dependent ALKBH5 promoter activation and GUDCA antagonism. Mutation of FXR sites abolished activity. **(I)** ChIP-qPCR confirming FXR binding to ALKBH5 promoter and reduction by GUDCA. Data are mean ± SD. *p < 0.05; **p < 0.01; ***p < 0.001; ns, not significant.

To elucidate the molecular mechanisms underlying GUDCA-induced glycolysis reprograming, we performed RNA-seq analysis of GUDCA-treated HCT8 cells. Among significantly downregulated genes, the ALKBH5 mRNA level was the most significantly downregulated in GUDCA-treated HCT8 cells. ALKBH5 is a well-known N^6^-methyladenosine (m^6^A) demethylase that plays a critical role in various cellular processes (Qu et al., 2022). m6A modification represents the most prevalent internal chemical modification in eukaryotic RNA. In mammals, m6A methylation is catalyzed by methyltransferases such as METTL3 and METTL14, while this modification is reversible and can be removed by the demethylases FTO or ALKBH5 (Jiang et al., 2021). Interestingly, our previous research revealed that bile acids regulate the m6A methylation of microRNA precursors in gastrointestinal tumors, thereby influencing cancer progression (Lin et al., 2020). To explore the potential involvement of m6A modification in GUDCA-mediated effects, we first examined the expression of m6A-related enzymes (METTL3, METTL14, WTAP, ALKBH5, and FTO) in HCT8 and SW480 cells following GUDCA treatment. The results demonstrated that GUDCA specifically suppresses ALKBH5 transcription and reduces its protein levels (Figures 4A,B), without affecting the transcriptional levels of other m6A-modifying enzymes (Figure 4A). Furthermore, clinical correlation analysis of serum GUDCA levels and ALKBH5 protein expression in paired CRC tissue specimens revealed a significant inverse relationship (r = −0.546, p < 0.001, Figure 4C), supporting the physiological relevance of this regulatory axis.

Current studies suggest that after bile acids are secreted into the intestine, both primary bile acids and secondary bile acids act as signaling molecules by binding to bile acid receptors. Different bile acids exert distinct effects on these receptors—some act as agonists, while others function as antagonists (Fuchs and Trauner, 2022). Therefore, we first compared the expression levels of various bile acid membrane receptors and nuclear receptors in HCT8 cells, and found that nuclear receptors, particularly FXR and VDR, showed the highest expression levels (Figure 4D). We then generated FXR- and VDR-knockout HCT8 cells to investigate whether they are involved in the transcriptional regulation of ALKBH5 by GUDCA. The results revealed that FXR deficiency abolished the effect of GUDCA on ALKBH5 transcription, indicating that GUDCA modulates FXR-mediated transcriptional regulation via an antagonistic mechanism (Figure 4E). In contrast, treatment with GW4064, a well-known FXR agonist, significantly upregulated ALKBH5 mRNA expression in both HCT8 and SW480 cells, opposing the effect observed with GUDCA (Supplementary Figure S1).

As a member of the nuclear receptor superfamily that functions as ligand-dependent transcription factors often forming heterodimers, FXR can regulate the transcription of target genes either directly or by recruiting other transcription factors (Kim et al., 2022). We thus analyzed the ALKBH5 promoter sequence and identified a potential FXR-binding site. Subsequently, we constructed luciferase reporter plasmids containing either the wild-type ALKBH5 promoter sequence or a mutant with the FXR-binding site disrupted (Figures 4F,G). Using both luciferase reporter assays and ChIP-qPCR, we demonstrated that GUDCA antagonizes FXR-mediated transcriptional regulation of ALKBH5 (Figures 4H,I). Taken together, these findings identify FXR as the key receptor through which GUDCA downregulates ALKBH5 transcription.

### ALKBH5 functions as a tumor suppressor in CRC

**FIGURE 5:**
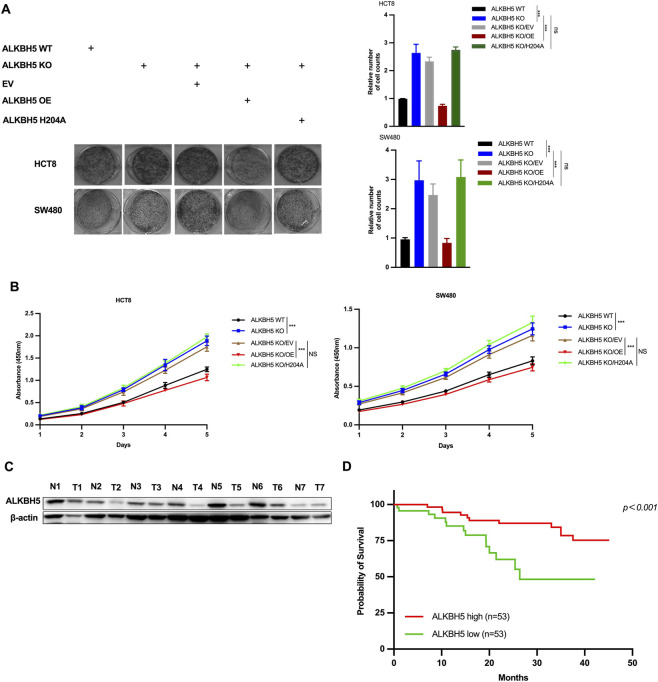
ALKBH5 Acts as a Tumor Suppressor to Restrain Colorectal Cancer Progression. **(A)** Colony formation assays showing ALKBH5 deletion enhanced proliferation, rescue with WT but not H204A reversed this effect in HCT8 and SW480 cells. **(B)** CCK-8 in cells with indicated genotypes. ***p < 0.001 vs. WT; ###p < 0.001 vs. KO. Scale bars = 1 cm. **(C)** I Western blots of ALKBH5 expression in paired CRC and normal tissues. ALKBH5 was reduced in tumors. **(D)** Kaplan-Meier curves for CRC patients with high (n = 53) versus low (n = 53) ALKBH5 expression. p < 0.001, log-rank test. Data are mean ± SD. *p < 0.05; **p < 0.01; ***p < 0.001; ns, not significant.

To investigate the role of ALKBH5 in CRC progression, we generated stable ALKBH5-knockout (ALKBH5-KO) and rescue cell lines expressing either wild-type ALKBH5 (ALKBH5-WT) or enzymatically inactive ALKBH5 H204A mutant. ALKBH5 deletion significantly enhanced HCT8 and SW480 cell proliferation and colony formation (Figures 5A,B). Rescue with wild-type ALKBH5, but not the H204A mutant, restored growth suppression, demonstrating that ALKBH5’s tumor-suppressive function requires its catalytic activity. Western blot analysis confirmed that both the wild-type and H204A mutant constructs were robustly expressed in ALKBH5-KO HCT8 cells at levels exceeding endogenous ALKBH5, ruling out the possibility that the functional failure of the H204A mutant was due to insufficient protein expression (Supplementary Figure S2).

In addition, analysis of CRC tissue microarrays revealed significantly reduced ALKBH5 expression in tumor tissues compared to adjacent normal mucosa (Figure 5C). Kaplan-Meier survival analysis showed that low ALKBH5 expression correlated with poor overall survival in CRC patients (Figure 5D), consistent with its tumor-suppressive role. These findings establish ALKBH5 as a tumor suppressor in CRC in a manner dependent on its intrinsic enzymatic activity and identify it as a critical downstream target of GUDCA signaling.

### ALKBH5 deficiency enhances ENO1 expression through increased mRNA translation

**FIGURE 6:**
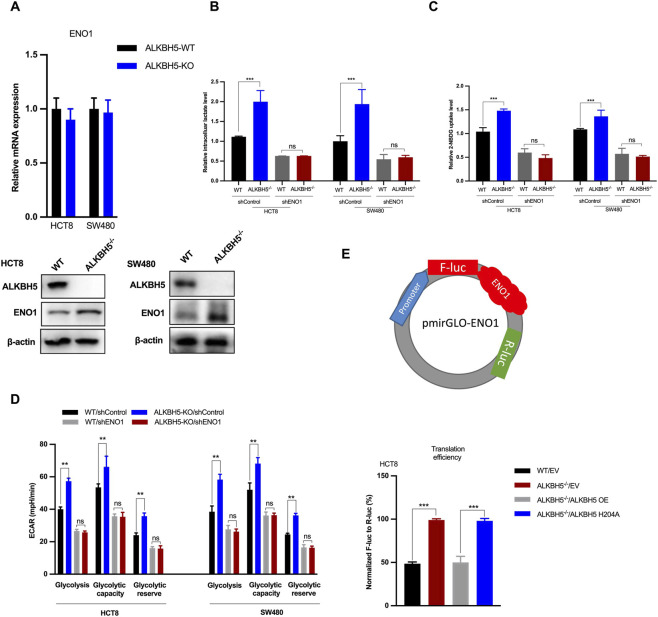
ALKBH5 Deficiency Augments ENO1 Expression Through Enhancing m6A-Mediated mRNA Translation Efficiency. **(A)** Western blots and qRT-PCR of ENO1 in WT and ALKBH5-KO HCT8/SW480 cells. ALKBH5 deletion increased ENO1 protein without affecting mRNA. ***p < 0.001; ns, not significant. **(B–D)** Lactate production **(B)**, glucose uptake 2-NBDG **(C)**, and ECAR profiles **(D)** in WT, ALKBH5-KO, shENO1, and ALKBH5-KO + shENO1 cells. ENO1 knockdown abolished ALKBH5-KO-induced glycolytic enhancement. **(E)** Luciferase reporter assays with pmirGLO-ENO1 construct in WT, ALKBH5-KO, KO + ALKBH5-WT, and KO + H204A cells. ALKBH5 deficiency enhanced ENO1 translation, WT but not H204A rescue reversed this. Data are mean ± SD. *p < 0.05; **p < 0.01; ***p < 0.001; ns, not significant.

Since GUDCA treatment reduces ALKBH5 expression while increasing ENO1 expression in colorectal cancer cells, it suggests that ALKBH5 may promote colorectal cancer glycolysis by influencing the key glycolytic enzyme ENO1. To investigate the underlying mechanism of ALKBH5, we first examined its effect on ENO1 expression. The results showed that ALKBH5 suppresses ENO1 expression at the protein level without affecting its transcriptional level (Figure 6A). We then established ENO1-knockdown (shENO1) HCT8 and SW480 colorectal cancer cells to assess the impact of ALKBH5 on glycolysis in both ENO1 wild-type and deficient contexts. The results demonstrated that ALKBH5 knockdown significantly increased intracellular lactate levels, 2-NBDG uptake, and ECAR in both HCT8 and SW480 cells compared to the control group. However, ENO1 knockdown abolished these changes (Figures 6B–D).

As a key enzyme in the glycolytic pathway, current research on ENO1 primarily focuses on its impact on the biological characteristics of tumors, with relatively few studies investigating the regulatory mechanisms controlling ENO1 expression (Sun et al., 2023). ALKBH5 is a well-known N6-methyladenosine (m6A) demethylase that plays important roles in cellular processes. It has been found that ALKBH5 can regulate the translational efficiency of various genes. Therefore, we generated ALKBH5-deficient HCT8 cells and rescued them with either wild-type ALKBH5 or the catalytically inactive ALKBH5 H204A mutant, followed by transfection with the pmirGLO-ENO1-WT plasmid. The results showed that ALKBH5 deficiency enhanced the translational efficiency of ENO1 mRNA, while rescue with wild-type ALKBH5, but not H204A mutant, suppressed translation (Figure 6E). These experimental results indicate that ALKBH5 reduces the translational efficiency of ENO1 mRNA in a manner dependent on its enzymatic activity.

### The GUDCA-ALKBH5 axis governs ENO1 m6A modification to drive mTOR activation

Recent studies have begun to focus on mRNA modifications of ENO1, including that m5C of ENO1 mRNA significantly impacts its expression (Chen et al., 2024). As a prevalent RNA modification, m6A influences RNA stability, translational efficiency, secondary structure, subcellular localization, alternative polyadenylation, and splicing, thereby playing critical roles in cellular processes (Gu et al., 2020). It has been recently reported that m6A reader proteins YTHDF1 and YTHDF3 facilitate the translation of m6A-modified mRNAs (Wang et al., 2022). However, whether m6A modification regulates ENO1 protein expression remains unexplored.

**FIGURE 7:**
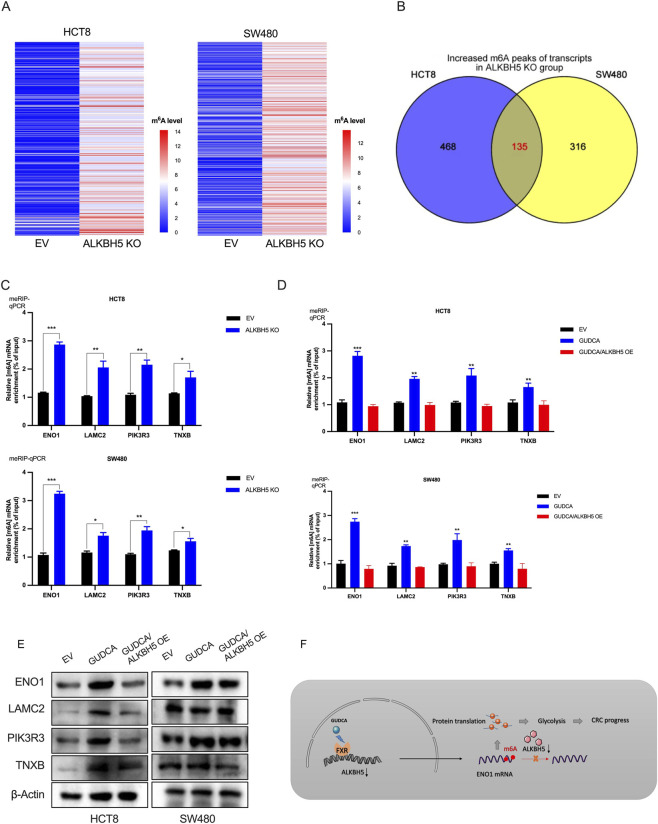
The GUDCA-ALKBH5 Axis Orchestrates ENO1 m6A to Activate mTORC1 Signaling and Metabolic Rewiring. **(A)** MeRIP-seq analysis showing global m6A hypermethylation in ALKBH5-KO HCT8 and SW480 cells. **(B)** Venn diagram of genes with altered m6A in both cell lines (135 overlapping genes including ENO1, LAMC2, PIK3R3, TNXB). **(C)** MeRIP-qPCR validation of m6A enrichment on ENO1, LAMC2, PIK3R3, and TNXB in WT and ALKBH5-KO cells. **(D)** MeRIP-qPCR showing GUDCA increased m6A on ENO1, LAMC2, PIK3R3, and TNXB; ALKBH5 overexpression reversed this effect. **(E)** Western blots and quantification of ENO1, LAMC2, PIK3R3, TNXB in cells treated with vehicle or GUDCA. GUDCA increased protein levels and mTORC1 activity, while ALKBH5 abolished this effect. **(F)** Schematic model illustrating the GUDCA-FXR-ALKBH5-ENO1-mTORC1 signaling axis in colorectal cancer. GUDCA antagonizes FXR to suppress ALKBH5 expression, resulting in ENO1 m6A hypermethylation and enhanced translation. Elevated ENO1 drives glycolytic reprogramming and mTORC1 activation, promoting CRC progression. Therapeutic targeting strategies are indicated. Data are mean ± SD. *p < 0.05; **p < 0.01; ***p < 0.001.

To investigate if ALKBH5 regulates ENO1 via an m6A-dependent mechanism, we established two stable ALKBH5-knockout human colorectal cancer cell lines and performed meRIP-seq analysis. The results showed that ALKBH5 significantly alters the m6A methylome in colorectal cancer (Figure 7A). A Venn diagram indicated that 135 genes exhibited consistent m6A modification changes in both knockout lines (Figure 7B). Further analysis revealed that ALKBH5 knockout led to a significant increase in m6A levels of ENO1 and key mTOR pathway genes, including LAMC2, PIK3R3, and TNXB. This regulatory effect was subsequently validated by meRIP-qPCR (Figure 7C).

We then treated colorectal cancer cells with GUDCA and performed meRIP-qPCR, which demonstrated that GUDCA markedly upregulates m6A modification of ENO1, LAMC2, PIK3R3, and TNXB. Importantly, overexpression of ALKBH5 reversed this effect (Figure 7D). Consistent with these findings, subsequent protein-level analysis confirmed the impact of m6A modification on the expression of ENO1, LAMC2, PIK3R3, and TNXB (Figure 7E)**.** Collectively, these results demonstrate that the GUDCA-ALKBH5 axis promotes m6A methylation on ENO1 and activates mTOR signaling (Figure 7F)**.**

## Discussion

This study unveils a previously unrecognized bile acid-orchestrated epitranscriptomic mechanism driving metabolic reprogramming in CRC. We demonstrate that GUDCA, a conjugated secondary bile acid elevated in CRC patients, functions as an oncometabolite by antagonizing FXR-mediated ALKBH5 transcription. The resulting ALKBH5 deficiency elevates m6A modification of ENO1 mRNA, enhancing its translation efficiency and glycolytic capacity. This GUDCA-ALKBH5-ENO1 axis subsequently activates mTORC1 signaling, establishing a feed-forward metabolic circuit that promotes CRC proliferation and progression. Our findings have important implications for understanding bile acid biology, epitranscriptomic regulation, and cancer metabolism.

Several lines of evidence support the pathogenic role of GUDCA in CRC. First, comprehensive bile acid profiling identified GUDCA as the most significantly elevated species in CRC patients, with levels correlating with tumor burden and poor prognosis. In addition, GUDCA demonstrated potent proliferative effects in CRC cells and xenograft models. Furthermore, therapeutic interventions targeting GUDCA or its downstream effectors showed significant anti-tumor efficacy in CRC cells. These findings extend previous observations that secondary bile acids promote colorectal carcinogenesis (Cong et al., 2024; Okumura et al., 2021) by identifying a specific conjugated bile acid species and elucidating its mechanistic basis. Interestingly, while ursodeoxycholic acid (UDCA) and its taurine conjugate (TUDCA) exhibit chemoprotective properties in CRC (Shah et al., 2006), our results indicate that GUDCA promotes tumorigenesis, highlighting the importance of bile acid conjugation patterns in determining biological activity.

The identification of FXR as the receptor mediating GUDCA’s effects on ALKBH5 represents a important expansion. in bile acid-FXR biology. Traditionally, FXR functions as a master regulator of bile acid homeostasis, activated by primary bile acids such as chenodeoxycholic acid (CDCA) (Liu Y. et al., 2025; Xu et al., 2023). Our finding that GUDCA antagonizes FXR to suppress ALKBH5 transcription reveals a novel mechanism whereby secondary bile acids can exert oncogenic effects through FXR inhibition. This is particularly relevant in CRC, where intestinal dysbiosis promotes the conversion of primary to secondary bile acids (Cai et al., 2022; Peng et al., 2024), potentially creating a tumor-permissive environment through FXR antagonism. Future studies should investigate whether other secondary bile acids exhibit similar FXR-antagonistic properties and whether FXR agonists could serve as chemopreventive agents in high-risk populations.

Our mechanistic dissection establishes ALKBH5 as a critical tumor suppressor in CRC whose loss drives metabolic reprogramming. While previous studies have reported context-dependent roles for ALKBH5 in different cancers—oncogenic in acute myeloid leukemia (Shen et al., 2020) but tumor-suppressive in glioblastoma (Goul et al., 2023)—our data clearly demonstrate a tumor-suppressive function in CRC. The enzymatic activity-dependence of ALKBH5’s growth-suppressive effects underscores the importance of m6A demethylation in restraining CRC progression (Tang et al., 2022). Importantly, our clinical data showing reduced ALKBH5 expression in CRC tissues and its correlation with poor survival further validate its tumor-suppressive role. These findings suggest that therapeutic strategies aimed at enhancing ALKBH5 expression or activity could represent a novel approach to CRC treatment.

The discovery that ALKBH5 regulates ENO1 translation through m6A modification adds a new layer of complexity to glycolytic enzyme regulation. While ENO1 overexpression has been widely reported in various cancers (Sun et al., 2023; Zhang T. et al., 2022), the mechanisms governing its post-transcriptional regulation have remained unclear. Our data demonstrate that elevated m6A modification in the ENO1 mRNA enhances its translation efficiency, independent of mRNA stability changes. Recent studies have shown that m6A can enhance translation through YTHDF1/3-dependent mechanisms (Li Q. et al., 2021) or even reader-independent structural changes (Zhang et al., 2020). Future structural and biochemical studies are warranted to precisely define how m6A modifications influence ENO1 mRNA translatability.

The mTORC1 activation downstream of GUDCA-ALKBH5-ENO1 signaling represents a critical convergence point linking metabolism to growth control. mTORC1 integrates diverse inputs, including growth factors, amino acids, and energy status, to regulate anabolic processes (Chen et al., 2021; Goul et al., 2023). Our findings demonstrate that GUDCA-induced glycolytic enhancement activates mTORC1, likely through increased ATP production and glycolytic intermediate accumulation. This creates a positive feedback loop wherein mTORC1 further stimulates glycolysis and proliferation (He et al., 2025), amplifying the oncogenic effects of GUDCA. Given that mTOR inhibitors have shown limited single-agent efficacy in CRC clinical trials (Zhang H. et al., 2022), our findings suggest that patient stratification based on bile acid profiles might identify subpopulations more likely to benefit from mTOR-targeted therapy.

The GUDCA–ALKBH5–ENO1 axis likely reshapes the TME toward immune evasion. ENO1-driven glycolysis elevates extracellular lactate, which suppresses CD8^+^ T cell cytotoxicity (Wang et al., 2023), stabilizes PD-L1 (Liang et al., 2026), and promotes M2 macrophage polarization (Lin et al., 2026). Although GUDCA-mediated FXR antagonism might partially relieve T cell inhibition (Liu S. et al., 2025), the glycolytic acceleration and PD-L1 stabilization driven by ENO1 upregulation would likely counteract this benefit. Functionally, this deleterious metabolic reprogramming phenocopies the glycolytic immune-evasion landscape associated with bile-acid signaling (Jiang et al., 2026), even though GUDCA operates through FXR antagonism rather than receptor stabilization. Notably, the immunomodulatory output of bile acids is not uniform but is dictated by the nuclear receptors each species engages: microbiota-derived metabolites such as 3-oxo-Δ4,6-LCA potentiate anti-tumor immunity through AR antagonism (Jin et al., 2025), highlighting the receptor-dependent nature of these effects. Pharmacological disruption of this axis may therefore sensitize CRC to immune checkpoint blockade, though rigorous *in vivo* investigation is needed to delineate its precise role in shaping anti-tumor immunity.

The tumor-promoting capacity of GUDCA observed *in vivo* cannot be fully understood without considering the crosstalk between local colonic signaling and systemic enterohepatic feedback. As a conjugated bile acid with limited passive intestinal absorption and low systemic bioavailability, oral GUDCA predominantly accumulates within the intestinal lumen, directly contacting orthotopic colonic tumors (Dawson et al., 2009). Yet, its broader repercussions engineered through enterohepatic routing are striking. By obstructing ileal FXR, GUDCA dismantles the canonical negative feedback brakes on bile acid production—principally via the FGF15/19 axis—thereby triggering an accelerated enterohepatic loop and a restructured global bile acid pool that, paradoxically, can drive up total systemic bile acid titers (Fleishman and Kumar, 2024). Such systemic realignments theoretically expand the regulatory reach of bile acids to FXR or membrane-bound counterparts like TGR5 across peripheral tissues and remote oncogenic niches (Fleishman and Kumar, 2024). Intriguingly, oral GUDCA retained its tumor-accelerating capacity even in a subcutaneous xenograft landscape where intraluminal contact is physically precluded. This phenomenon implies that either trace circulating echelons of GUDCA possess enough potency to directly block tumor-localized FXR, or the newly reconfigured systemic bile acid landscape acts as a potent distal oncogenic driver. Disentangling these localized cell-autonomous circuits from macro-level enterohepatic feedback networks persists as a formidable bottleneck in bile acid pathophysiology.

Several limitations of our study warrant discussion. Several limitations of our study warrant discussion. First, our clinical findings are preliminary, as the small cohort (n = 106) and low events-per-variable ratio (5.2) limit the statistical power of the survival analysis. Second, our current *in vivo* models cannot distinguish whether GUDCA promotes tumor growth via localized receptor signaling or through broader systemic disruptions in global bile acid homeostasis. Overcoming this requires future tissue-specific knockouts and detailed GUDCA–FXR structural binding studies. Furthermore, the precise m6A reader proteins mediating ENO1 translation enhancement remain incompletely characterized. Finally, large-scale cohorts and clinical trials are ultimately essential to validate these findings and assess translational safety in CRC patients.

In conclusion, our study identifies GUDCA as a critical oncometabolite that drives CRC progression through an epitranscriptomic mechanism involving FXR-ALKBH5-mediated m6A modification of ENO1. This work reveals unexpected connections among bile acid signaling, RNA modification, and metabolic reprogramming, establishing new paradigms in cancer biology. Our findings not only advance mechanistic understanding of CRC pathogenesis but also identify multiple therapeutic vulnerabilities with translational potential. Future studies should investigate whether similar bile acid-epitranscriptome-metabolism axes operate in other gastrointestinal malignancies and whether interventions targeting these pathways can improve outcomes for cancer patients.

## Materials and methods

### Clinical materials

This study enrolled CRC patients who underwent surgical resection at Fujian Cancer Hospital between 2020 and 2022. The study protocol was approved by the Ethics Committee of Fujian Cancer Hospital, and written informed consent was obtained from all participants. The inclusion criteria were: (1) histologically confirmed primary colorectal adenocarcinoma; (2) underwent radical (R0) resection; (3) availability of complete clinicopathological and follow-up data; and (4) adequate tumor tissue specimens for molecular analysis. Patients were excluded if they met any of the following criteria: (1) history of other primary malignancies; (2) receipt of neoadjuvant chemotherapy or radiotherapy; (3) presence of severe hepatobiliary diseases; (4) perioperative death (within 30 days after surgery) or loss to follow-up; or (5) poor quality of tissue specimens.

### Cell culture

Human CRC cell lines HCT8 and SW480 were described in previous studies (Yang et al., 2024). Briefly, cell were cultured in RPMI-1640 medium supplemented with 10% fetal bovine serum (FBS), 100 U/mL penicillin, and 100 μg/mL streptomycin at 37 °C with 5% CO2. Cells were authenticated by short tandem repeat profiling and routinely tested for *mycoplasma* contamination.

### Cell treatment

Human colorectal cancer cell lines were were seeded in appropriate culture plates and allowed to adhere overnight. The following day, cells were treated with 50 μM GUDCA. This concentration of GUDCA was chosen to approximate the median plasma levels observed in colorectal cancer patients (median 55.5 μM; 25th-75th percentile: 30.9–111.0 μM) as reported in a previous study (Kühn et al., 2020). Control cells received an equivalent volume of vehicle (PBS) and were processed in parallel. All experiments were performed in triplicate and repeated at least three times independently.

### Bile acid profiling

Serum bile acids were quantified using UHPLC-MS/MS as previously described (Lin et al., 2020). Briefly, serum samples were extracted with acetonitrile containing internal standards, and bile acids were separated and detected using electrospray ionization.

### RNA extraction and quantitative real-time PCR

qRT-PCR were described in previous study (Lin et al., 2020). Total RNA was extracted using TRIzol reagent and reverse-transcribed using PrimeScript RT Kit. qRT-PCR was performed using SYBR Green Master Mix. Gene expression was normalized to GAPDH using the 2^-ΔΔCt^ method. Primer sequences are provided in Supplementary Table S1.

### Western blotting

Cells were lysed in RIPA buffer containing protease and phosphatase inhibitors. Protein concentrations were determined using BCA assay (Pierce). Equal amounts of protein were separated by SDS-PAGE and transferred to PVDF membranes. After blocking with 5% BSA, membranes were incubated with primary antibodies overnight at 4 °C, followed by HRP-conjugated secondary antibodies. Proteins were visualized using ECL reagent (Millipore).

### Metabolomic profiling

HCT8 cells treated with GUDCA or vehicle control were harvested, and metabolites were extracted using cold methanol. Untargeted metabolomic analysis was performed using LC-MS/MS.

#### MeRIP-sequencing

m6A-modified RNA was immunoprecipitated using anti-m6A antibody as previously described (Lin et al., 2020). Input and immunoprecipitated RNA were subjected to library preparation and sequencing on Illumina platform. Differential m6A peaks between groups were identified using DESeq2 (|log2FC| > 1.5, FDR < 0.05).

#### Glycolytic assays

Glycolytic function was assessed by measuring intracellular lactate concentration, 2-NBDG glucose uptake, and extracellular acidification rate (ECAR) using a Seahorse XF Analyzer with sequential metabolic challenge. All measurements were normalized to total protein content.

#### Luciferase reporter assays

ALKBH5 promoter or ENO1 5'/3′UTR sequences were cloned into pGL3-Basic or pmirGLO vectors. HEK293T cells were co-transfected with reporter plasmids, expression vectors, and control using Lipofectamine 3,000. Luciferase activities were measured 48 h post-transfection using Dual-Luciferase Reporter Assay System (Promega) and normalized to Renilla activity.

### Chromatin immunoprecipitation (ChIP)-qPCR

ChIP assays were performed as previously described (Lin et al., 2020). Briefly, HCT8 cells were cross-linked with 1% formaldehyde, lysed, and chromatin was fragmented by micrococcal nuclease digestion. Chromatin was immunoprecipitated using anti-FXR antibody or IgG control, and enrichment at ALKBH5 promoter was quantified by qPCR. Primer sequences are provided in Supplementary Table S1.

### Animal studies

All animal experiments were approved by the Institutional Animal Care and Use Committee of Fujian cancer hospital affiliated to Fujian medical University. For subcutaneous xenograft models, 5 × 10^6^ HCT8 cells were injected into the flanks of 6-week-old male BALB/c nude mice. One week after injection, mice were randomly allocated to control and treatment groups. Mice in the treatment and control groups were subsequently administered either GUDCA (100 mg/kg/day, Sigma-Aldrich; a dosage based on previous literature (Chen et al., 2023)) or its vehicle, respectively, via daily oral gavage. GUDCA was dissolved in a solution of 2% DMSO, 48% PEG 400, and 50% H_2_O. All mice were sacrificed and tumors were embedded in paraffin for tissue staining.

### Statistical analysis

Data are presented as mean ± standard deviation (SD) from at least three independent experiments. Statistical comparisons were performed using unpaired two-tailed Student’s t-test for two groups or one-way ANOVA with Tukey’s post-hoc test for multiple groups. Kaplan-Meier survival analysis was performed with log-rank test. Multivariate Cox regression was used to assess prognostic factors. Correlations were assessed using Pearson or Spearman correlation coefficients. The proportional hazards assumption was verified using Schoenfeld residuals, and model fit was assessed by the likelihood-ratio test. All analyses were performed using R software. P < 0.05 was considered statistically significant (*P < 0.05, **P < 0.01, ***P < 0.001).

## Data Availability

The original contributions presented in the study are included in the article/Supplementary Material, further inquiries can be directed to the corresponding authors.

## Supplementary material

SUPPLEMENTARY FIGURE S1 Effect of FXR agonist GW4064 on ALKBH5 mRNA expression in CRC cells.

SUPPLEMENTARY FIGURE S2 Validation of wild-type and H204A mutant ALKBH5 protein expression in ALKBH5-KO HCT8 cells.
