# Supplementary figures and images for: GUDCA drives colorectal cancer progression via ALKBH5-mediated m6A modification of ENO1 and glycolytic reprogramming
Source: Front Pharmacol. 2026 Jul 10;17:1791014. doi: 10.3389/fphar.2026.1791014 (PMC13395929; doi:10.3389/fphar.2026.1791014)

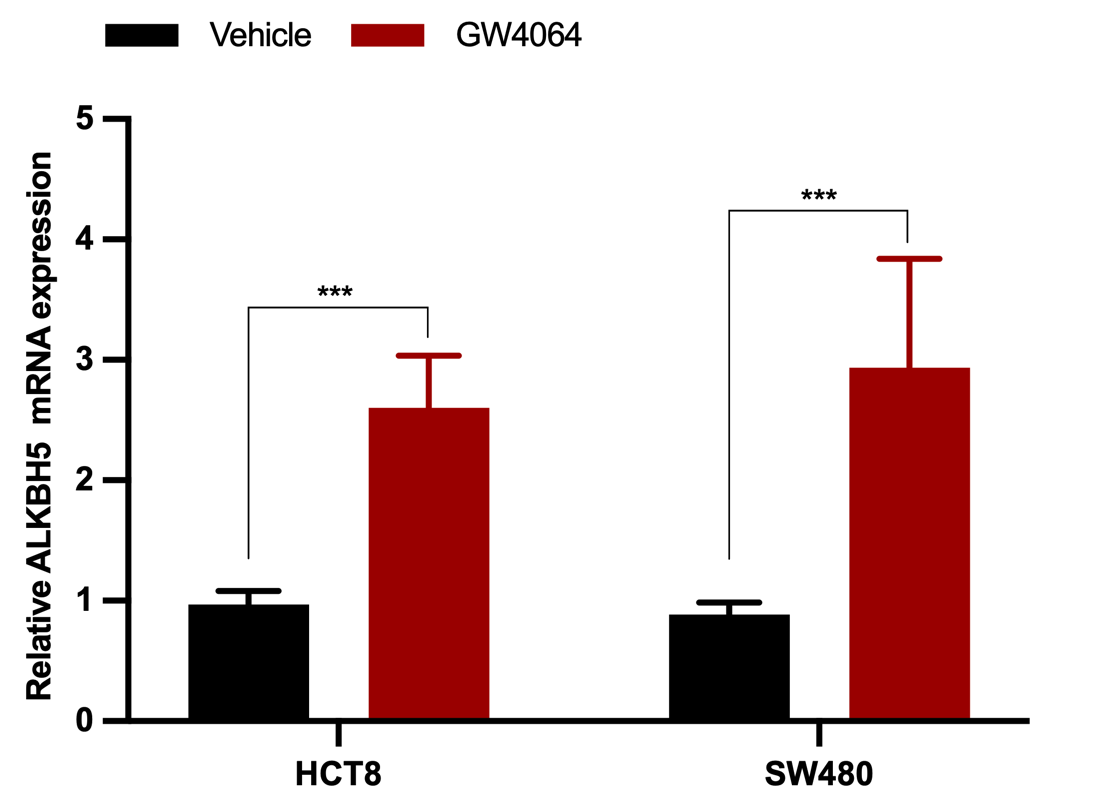

Supplement: Supplementary file 1 [file Image1.tiff]

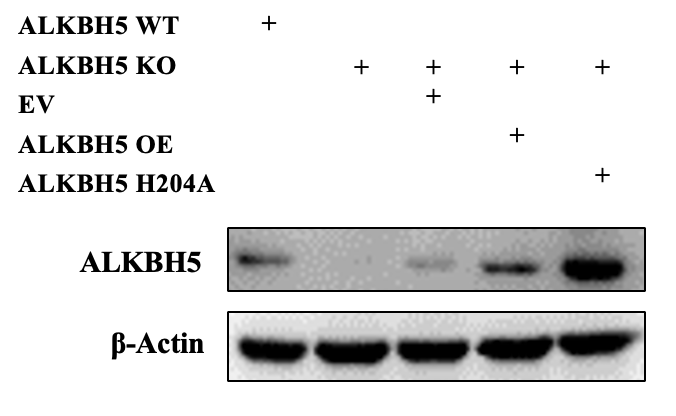

Supplement: Supplementary file 3 [file Image2.tif]
