# Supplementary material for: GUDCA drives colorectal cancer progression via ALKBH5-mediated m6A modification of ENO1 and glycolytic reprogramming
Source: Front Pharmacol. 2026 Jul 10;17:1791014. doi: 10.3389/fphar.2026.1791014 (PMC13395929; doi:10.3389/fphar.2026.1791014)
Supplement: Supplementary file 2 [file Table1.docx]

Supplementary Table 1. Sequence of primers, shRNA or probe used in this study.

| qPCR primers for mRNAs | | |
| --- | --- | --- |
| Gene | Forward | Reverse |
| ENO1 | CCTCAACAGTGGTTCTCTCTAAC | AGACACCTGGAAGGAACAATC |
| PKM2 | ATTATTTGAGGAACTCCGCCGCCT | ATTCCGGGTCACAGCAATGATGG |
| LDHA | GAAGACTCTGCACCCAGATTTA | TCACCTCATAAGCACTCTCAAC |
| METTL3 | AAGCTGCACTTCAGACGAAT | GGAATCACCTCCGACACTC |
| METTL14 | AGAAACTTGCAGGGCTTCCT | TCTTCTTCATATGGCAAATTTTCTT |
| WATP | GGCGAAGTGTCGAATGCT | CCAACTGCTGGCGTGTCT |
| ALKBH5 | CTCAAGTCGGGACTGCATAAT | CACACATATCAGGGCGAAGT |
| FTO | GAAGACACTTGGCTCCCTTATC | CAGATACACTGCTGGCTTCTC |
| LAMC2 | CAACTGAGGTTCTTGGGATACA | GTTGACCTGAGCATACCCATTA |
| PIK3R3 | AGCAGACATCCTTGGTTCAG | CTCTCTTCCCACTTCCTCTTTATC |
| TNXB | GGTATTCACCCACCGCATTA | CCAACTCCTCCAGGATCTCTA |
| β-Actin | CCAACCGCGAGAAGATGA | CCAGAGGCGTACAGGGATAG |

| Sequence of shRNA and sgRNA | |
| --- | --- |
| shENO1 | CGGCGTTCAATGTCATCAATG |
| h-sg-FXR | TTGGCTGAATGTATGTATAC |
| h-sg-VDR | GGGGTCGTAGGTCTTATGGT |
| h-sg-ALKBH5 | GATCAACGACTACCAGCCCGG |

| Primers for MeRIP-qPCR | |
| --- | --- |
| ENO1-Forward | GGTATCTATGAGGCCCTA |
| ENO1-Reverse | TCAATCTTCTCTTGTTCT |
| LAMC2-Forward  LAMC2-Reverse  PIK3R3-Forward  PIK3R3-Reverse  TNXB -Forward  TNXB -Reverse | TGACAACTCCGGACGGTG  GATGCCAGCTGGGTCACA  TGCTCGGCCTCTCCACTTCACA  ACGTCAGGCTTGCCTACCCTGT  AGACAGATGTGCGGACCC  GACCCCGAGGCTGAGGGT |
